# Future Challenges and Opportunities of Extracellular Matrix Hydrogels in Female Reproductive Medicine

**DOI:** 10.3390/ijms23073765

**Published:** 2022-03-29

**Authors:** Emilio Francés-Herrero, Adolfo Rodríguez-Eguren, María Gómez-Álvarez, Lucía de Miguel-Gómez, Hortensia Ferrero, Irene Cervelló

**Affiliations:** 1 Department of Pediatrics, Obstetrics and Gynecology, School of Medicine, University of Valencia, 46010 Valencia, Spain; emilio.frances@ivirma.com; 2 Fundación IVI, IVI-RMA Global, 46026 Valencia, Spain; adolfo.rodriguez@ivirma.com (A.R.-E.); maria.gomez@ivirma.com (M.G.-Á.); lucia.demiguel@ivirma.com (L.d.M.-G.); hortensia.ferrero@ivirma.com (H.F.); 3 Reproductive Medicine Research Group, IIS La Fe, 46026 Valencia, Spain

**Keywords:** bioengineering, ECM hydrogels, reproductive medicine, female tract

## Abstract

Bioengineering and reproductive medicine have progressed shoulder to shoulder for several decades. A key point of overlap is the development and clinical translation of technologies to support reproductive health, e.g., scaffold-free constructs, polymeric scaffolds, bioprinting or microfluidics, and hydrogels. Hydrogels are the focus of intense study, and those that are derived from the extracellular matrix (ECM) of reproductive tissues and organs are emerging as promising new players given their results in pre-clinical models. This literature review addresses the recent advances in the use of organ-specific ECM hydrogels in reproductive medicine, considering the entire female reproductive tract. We discuss in-depth papers describing the development of ECM hydrogels, their use in in vitro models, and their in vivo application in preclinical studies. We also summarize the functions of hydrogels, including as grafts, carriers for cell transplantation, or drug depots, and present the potential and possible scope for use of ECM hydrogels in the near future based on recent scientific advances.

## 1. Introduction

Male and female factors have an equal impact on infertility, defined as the inability to achieve a clinical pregnancy despite regular unprotected sexual intercourse for one year [1]. However, as per the available literature and our research background, this review for the special issue of the International Journal of Molecular Sciences (Smart Hydrogels and Hydrogels Composites: Current Challenges and Opportunities) will focus on the female reproductive system. The female reproductive system consists of four main organs: the ovaries, or female gonads; the oviducts, also called fallopian tubes, which connect the ovaries and the uterus and where fertilization occurs; the uterus, responsible for embryo implantation and fetal development; and the vagina, which connects the uterine cervix to external structures and is essential for intercourse and newborn delivery [2].

Clinical approaches to conditions of the female reproductive system increasingly leverage bioengineering to provide not only stand-alone treatments but also enhancers or carriers for support or management of diseases (endometriosis, adenomyosis, or infections), disorders (premature ovarian failure, endometrial or vaginal atrophy, or uterine polyps), syndromes (Asherman or Mayer–Rokitansky–Küster-Hauser), or even tissue preservation processes (ovarian cortex and oocyte cryopreservation) [3]. Bioengineering has seen incredible progress driving many technological advances in biological and medical sciences, which have then been translated into constant advances in the gynecological field [4].

Hydrogels, in particular, are a promising and remarkable technology. Hydrogels are three-dimensional (3D) microenvironments consisting of cross-linked polymers with above 90% water content [5]. These water-based scaffolds are valuable due to their characteristic malleability and non-adhesive nature that allows easy application, e.g., as injectable forms [6]. Also, hydrogels can support cell/tissue proliferation and growth, which is key for tissue regeneration therapies [5]. Hydrogels are classified according to features such as ionic charge, biodegradability, cross-linking, physical properties, biochemical responses, or preparation methods [7], as well as by their origin: natural, synthetic, or hybrid [7]. Hydrogels are applicable for different purposes, including treatment delivery (e.g., of proteins, drugs, or stem cells), tissue environment mimicry, or tissue regeneration.

Hydrogels entered use in the gynecological field in the 20th century with initial reports coming from animal models. The tested applications include the use of collagen hydrogels for carrying stem cells or growth factors in murine models of intrauterine adhesions [8,9], premature ovarian failure [10,11], and vaginal atrophy [12]. Other natural hydrogels, for example those that are composed of alginate, can mimic the native microenvironment before murine ovary cryopreservation [13,14]. The value of these natural hydrogels lies in their biocompatibility, biodegradability, non-immunogenicity, and non-toxicity that is intrinsic to these biomaterials. However, these natural materials have limitations based mainly on their mechanical properties (e.g., time of gelation, deformation, or elasticities) [15]; thus, some approaches combine hydrogels of natural and synthetic origins [16].

The 3D native cellular environment that hydrogels aim to recreate is more complex than scaffolds that are just composed of collagen, hyaluronic acid, or synthetic alternatives. This surrounding milieu is called the extracellular matrix (ECM), which is defined as a non-cellular 3D network that is composed of a wide variety of macromolecules (collagens, elastin, proteoglycans, glycosaminoglycans (GAGs), or laminins, among others) that provide structural and functional support [17,18]. The types of molecules in the matrix as well as their interactions affect cell behaviors and other biological processes (e.g., GAGs present growth factors to cell receptors, the cross-linked matrix releases chemical and paracrine signals, etc.) [19]. Thus, the use of decellularized native ECM emerged as a strong alternative to simpler natural or synthetic hydrogels. Decellularized ECM is responsible for supporting the complex network in which different tissues and cells organize, providing a 3D structure and participating in the homeostasis of and interactions and adhesions between the cells/tissues and their microenvironmental niche [17].

The decellularization process (very well described in the last years [20]) is crucial for obtaining functional ECM. Organ decellularization is achieved through physical (e.g., freezing and thawing, agitation, or pressurization), chemical (e.g., detergents, solvents, and ionic solutions), or enzymatic (e.g., trypsin or collagenase) methods that are designed to preserve protein scaffold structure while removing the cells and consequently the antigenic epitopes. This is one of the most used techniques for producing native hydrogels [18], and the process demonstrates success for many organs, e.g., heart [21], liver [22], pancreas [23], and kidney [24]. The physical and chemical properties of decellularized ECM hydrogels are crucial to fulfill their clinical functions. These characteristics depend not only on the native tissue and its mechanical properties, but also on the decellularization approach or terminal sterilization methods. Thus, a proper and accurate characterization of this type of biomaterial is key before its application [25].

ECM hydrogels have emerging applications in the field of gynecology and promising preclinical results. Accordingly, this literature review provides a global overview of ECM hydrogels, including research describing their development and use in in vitro models and their in vivo application in preclinical studies. We address recent advances in the use of organ-specific ECM hydrogels in reproductive medicine, considering studies that span the entire female reproductive tract. Finally, we cover the potential and possible scope of ECM hydrogels for their application in the near future.

## 2. ECM Hydrogels, an Overview

### 2.1. Conceptualization and Historical Context

The first report of a crude decellularization technique in 1948 [26] led to the design and implementation of many decellularization protocols for virtually all tissues and organs [27,28]. The common goal of all such techniques is to achieve efficient cell and epitope removal, while best preserving the native composition and structure of the ECM. Work in the 1980s broke ground with the use of acellular small intestinal submucosa (SIS) as a bioscaffold for tissue reconstruction [29]. Since then, autologous and xenogeneic decellularized ECM-derived materials have attained approval for use in humans by national health agencies, and numerous preclinical and clinical studies demonstrate the safety and efficacy of these materials for a variety of tissues, such as the skeletal system, the skin, and the heart [30].

ECM scaffolds (solid native matrices) can become hydrogels by solubilization followed by neutralization and gelling—a discovery that expanded their in vitro potential and in vivo application. ECM hydrogels retain the tissue-specific bioactivity that characterizes ECMs and provide a polymeric structural support that is capable of guiding and maintaining cellular behavior [31]. They also retain the bioinductive, antimicrobial, chemoattractive, and immunomodulatory properties that are associated with specific proteins such as collagen. In addition, ECM hydrogels offer key advantages over ECM-derived solid scaffolds, including their homogeneous concentration, ability to be delivered by minimally invasive procedures (e.g., injection as a pre-gel solution via needle or catheter), or their ability to gel in situ and conform to laboratory vessels or irregular body cavities.

The first ECM hydrogels were derived from SIS [32] in 1998. These hydrogels and those that are derived from urinary bladder matrix are the most well-characterized and utilized [33,34]. However, in recent years, dozens of scientific reports have described the use of ECM hydrogels in almost every organ system. Indeed, these materials have utility in preclinical applications for the treatment of traumatic brain injury [35], ulcerative colitis [36], and stroke [37]. Thus, their application to reproductive systems, while perhaps unsurprising, promises exciting opportunities.

### 2.2. How to Make Hydrogels from ECM?

**Figure 1:**
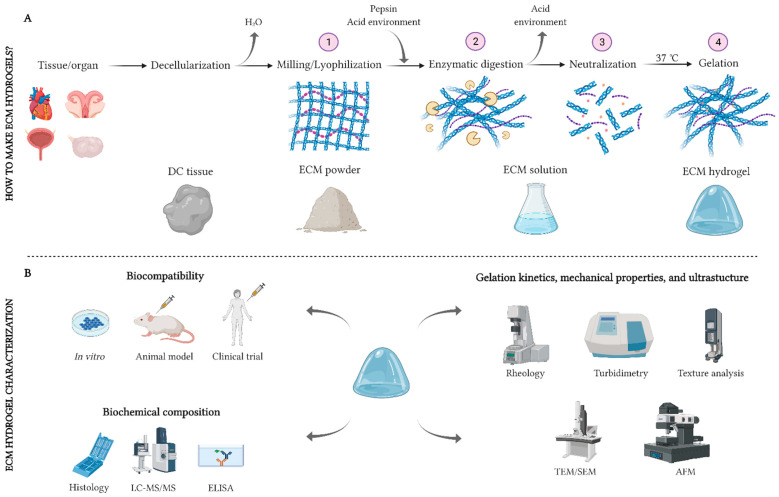
Schemas for generating and characterizing an ECM hydrogel. (**A**): The procedure for decellularizing, solubilizing, and gelling a native ECM. (**B**): Main techniques for the biological, biochemical, and physicomechanical characterization of ECM hydrogels. AFM: atomic force microscope; ECM: extracellular matrix; ELISA: enzyme-linked immunosorbent assay; LC-MS/MS: liquid chromatography tandem-mass spectrometry; SEM: scanning electron microscope; TEM: transmission electron microscope. Section A is adapted with permission from López-Martínez et al. 2021 [40]. Created with Biorender.com.

The formation of an ECM hydrogel involves the solubilization of the ground decellularized ECM material into monomeric protein components (also known as digestion), followed by pH- and/or temperature-controlled neutralization and induction of re-aggregation of the monomeric components (Figure 1A). The solubilization of powdered ECM by the enzymatic action of pepsin in a solution of hydrochloric [33] or acetic [32] acid is the most prevalent method for the formation of an “ECM pre-gel” solution. Depending on the clinical application, periods of 24–96 h are used to obtain a solution; then, the liquid is neutralized at physiological pH and saline concentration and a fully functional “ECM hydrogel” is obtained when the temperature is raised to physiological values (37 °C).

Solubilization of the ECM components also can occur by a physical homogenization process in a high-salt buffer with a mortar and pestle or high-speed shear mixer. This physical treatment may be preceded by an enzymatic digestion step with dispase and is usually followed by one or more extraction steps with urea [38,39].

#### 2.2.1. ECM Hydrogel Polymerization: The Role of pH, Temperature, and Cross-Linking Methods

Since native collagen is the major component of ECM hydrogels, the polymerization kinetics of ECM and purified collagen solutions share common features. The gelation characteristics of collagen are well-described [41], but the presence of other biomolecules and polymers in ECM hydrogels could provide unique viscoelastic properties. The fact that fibrillar collagen polymerization is inducible by changes in pH or temperature gives ECM hydrogels significant advantages over other natural or synthetic hydrogels. This aspect makes them ideal candidates for in vivo use since they gel in situ once they are administered. Interestingly, this temperature-dependent process is reversible [42].

No exogenous cross-linking agents are required for the formation of ECM hydrogels, preculding toxicity or irradiation risks [43]. However, various cross-linking techniques have been developed to improve their mechanical properties. They aim to stabilize the polymeric structure of collagen by forming covalent bonds, maintaining its native integrity and conformation. This process is based on the conjugation of reactive groups, e.g., carboxylate (─COO─) and amine (─NH2), of the collagen molecule with reactive cross-linkers, e.g., glutaraldehyde [44] or gepinin [43]. Cross-linking improves hydrogels’ mechanical properties, increasing their resistance to chemical and enzymatic degradation. This step also reduces their immunogenicity and sterilizes the hydrogels.

#### 2.2.2. Importance of the Native Tissue

The ECM that is secreted by the cells of each tissue and organ has a unique and changing composition, with crucial effects on cell morphogenesis, differentiation, and homeostasis. Thus, tissue/organ-specific ECM-derived materials have variable mixtures of biomolecules with a proven influence on the mechanical and biochemical properties of the material. This provides an exclusive and specific milieu that is related to the native origin of the ECM. In the case of hydrogels, this has a direct impact not only on their composition and underlying mechanisms, but also on their gelation, viscosity, or degradation properties [45]. Extensive characterization of these variables as well as assessment of biocompatibility is necessary to support the use of ECM hydrogels.

### 2.3. ECM Hydrogel Characterization

#### 2.3.1. Biocompatibility

Allogenic and xenogeneic antigens are recognized as foreign by immunocompetent hosts and can induce a pro-inflammatory response resulting in rejection of the tissue [46]. One of the main advantages of ECM derivatives is the high-degree of species conservation of the ECM components, making them tolerable even by xenogeneic recipients [47,48]. In most cases, the biocompatibility of ECM scaffolds and hydrogels is first evaluated in vitro (Figure 1B) [49]. In vitro assays can evaluate cell survival, proliferation, and differentiation in the specific hydrogel microenvironment and the hydrogel degradation rate. Recellularization assays, culture media supplementation, cell encapsulation, and coatings are commonly employed strategies to assess the biocompatibility of hydrogels [50,51,52]. Subsequently, their immunomodulatory capacity can be evaluated in animal models (e.g., by subcutaneous injections) [52]. In these assays, it is useful to assess the infiltration of immune cells into the xenografts. A high inflammatory response can indicate rejection, but ECM hydrogels have also been found to promote a regulatory macrophage activation state, which is associated with remodeling in vivo [53]. If the safety of ECM products is proven and accepted by a public health administration, clinical trials can be used to evaluate, in-depth, their therapeutic potential in humans [21].

#### 2.3.2. Biochemical Composition

The ECM is composed of a complex mixture of structural and functional biomolecules that can mostly be retained after decellularization and solubilization if appropriate methods are used. Collectively referred to as the “matrisome”, the proteins of the extracellular environment include collagens, proteoglycans, glycoproteins, secreted factors, ECM regulators, and ECM-associated proteins [54]. Mass spectroscopy is one of the most widely exploited techniques to biochemically characterize ECM hydrogels (Figure 1B). Specifically, reverse-phase high-performance liquid chromatography combined with tandem mass spectroscopy (RPLC-MS/MS) supports determination of the proteomic profile of ECM hydrogels [55,56]. Other methods to study the composition of ECM hydrogels include immunohistochemistry techniques, DNA and colorimetric quantification tests, or ELISA assays [28].

#### 2.3.3. Gelation Kinetics, Mechanical Properties, and Ultrastructure

It is not only the bioactivity and biocompatibility of ECM hydrogels that determines their utility. Their viscoelastic and mechanical properties also influence their in vivo (injectability, absorption, in situ gelation) and in vitro (stiffness-related cell viability, proliferation, and differentiation) behaviors. In general, ECM hydrogels are thermosensitive viscoelastic materials that exhibit mechanical properties similar to a viscous liquid at low temperatures and mechanical properties that are intermediate between that of a solid and liquid at physiological temperatures (37 °C) [55]. The gelation kinetics and viscoelastic response of ECM hydrogels or pre-gel solutions can be measured by rheology, indentation, texture analysis, or turbidimetric techniques [49,52,57].

The conversion of solid ECM scaffolds into ECM hydrogels leads to the loss of the native 3D conformation. However, the complex fiber network of hydrogels retains the ability to guide and permit specific cellular behaviors via contact guidance and fibril-bound proteins that interact with membrane integrin receptors. The fiber diameter, pore size, and fiber orientation are key variables in this interaction event [38]. Scanning electron microscopy (SEM) can be considered the most suitable technique to visualize the topology of hydrogels and analyze their ultrastructural properties, but transmission electron microscopy (TEM) [51], atomic force microscopy (AFM) [58], and confocal microscopy [59] are also applicable.

### 2.4. A Step Further: Hybrid Hydrogels

Recent efforts combine ECM hydrogels with other natural or synthetic hydrogels to enhance their mechanical properties and biological activity [60,61]. For example, it has been reported the use of liver-derived ECM:collagen, ECM:hyaluronic acid (HA), or ECM:heparin-conjugated HA hybrid hydrogels for 3D in vitro maintenance of hepatocyte function [61]. Other approaches utilize particulate forms of ECM materials as additives to synthetic hydrogels [62] or apply ECM hydrogels as coatings for solid biomaterials [63].

## 3. Deriving ECM Hydrogels from Reproductive Organs

ECM products that are obtained from decellularization of female reproductive organs have salient roles in the field of reproductive medicine, with research focusing mainly on those that are derived from the uterus [51,64,65,66,67,68,69,70,71,72,73,74,75,76,77,78,79,80], ovary [50,81,82,83,84,85,86,87,88,89,90,91,92], cervix [93], vagina [94,95], and placenta [96,97,98,99,100,101,102,103,104,105]. The variable methods that are used for decellularization are dependent on the tissue type and its characteristics. Tissue fragment immersion and whole organ perfusion are the most widely used in the reproductive field. ECM scaffold characterization is more standardized than decellularization, making use of the usual histological, immunohistochemical, and electron microscopy techniques (Table S1).

Recent work converted decellularized scaffolds from reproductive organs into ECM hydrogels (Table 1), which, as discussed in Section 2.1, have the main advantage of being able to conform to the shape of any defect site, since they are first injected as a viscous fluid (pre-gel) and finally polymerize into a 3D structure (hydrogel) at physiological temperature [55]. The development of ECM hydrogels from the female reproductive tract is still nascent, but promising results include the formation of hydrogels from decellularized uterus [40,52,106,107], ovary [108,109], oviduct [110], vagina [111], and placenta [112,113,114] (Table 1).

### 3.1. Endometrial Hydrogels

The uterus is the largest organ of the female reproductive system in most mammals and is responsible for supporting gestation. It is made up of three anatomical layers—perimetrium, myometrium, and endometrium—but bioengineering approaches have particularly focused in the endometrial layer due to its implication and importance in gynecological pathologies and embryo implantation.

A large number of studies have isolated whole murine uterine ECM due to their high availability, small size, and suitability for subsequent studies with in vivo rat and mouse models (Table S1). However, some groups have worked with higher mammals such as rabbits, sheep, or pigs in an attempt to fine-tune decellularization of uteri with a more human-like size. Remarkably, the isolation and decellularization of uterine tissues with different and specific compositions and functionalities (myometrium and endometrium) have already been applied in rats, rabbits, and humans (Table S1). Indeed, uterine ECM hydrogels are usually obtained by the decellularization of the endometrium (EndoECM) [40,52,106,107].

Whole uterine perfusion is the most commonly used decellularization procedure to generate EndoECM. The large size of the uterine arteries facilitates their canalization and connection to peristaltic pumps, which are responsible for pushing detergents and enzymatic agents into the organ. Once the uterus is decellularized, the acellular endometrium can be isolated mechanically [107]. Other methods, such as immersion and agitation, require cutting of the endometrium, which is not easily distinguishable, before decellularization. This demands a high degree of expertise [73,74].

In 2019, Campo et al. described, for the first time, the isolation of an ECM hydrogel from decellularized rabbit endometrium. To achieve this, the acellular endometrium was separated via microdissection under a stereomicroscope, lyophilized, and milled. Then, this powder was solubilized via a modified version of the Freytes protocol [33]. After extensive characterization, the hydrogels were used to create biological surface coatings for embryo culture. Interestingly, functionalized culture plates influenced early embryonic development demonstrating that ECM hydrogels contain tissue-specific proteins that can positively impact cell culture [107]. This fact is particularly relevant in dynamic tissues, such as the human endometrium, which undergoes major cyclical changes in its proliferation and secretion phases [107].

These decellularization and solubilization methods were later replicated by López-Martínez et al. using a pig uterus. In an initial study, different types of endometrial cells were cultured in 2D and 3D conditions using EndoECM hydrogels. Subsequently, a 3D endometrium-like co-culture system made of epithelial and stromal cells was developed. Also, the in vivo biocompatibility of EndoECM was demonstrated in a subcutaneous murine model [52]. Based on these results, this group later injected EndoECM hydrogels into a murine model of endometrial damage. Their purpose was to use these hydrogels as growth factor (GF) carriers due to the ability of hydrogels to bind biomolecules and gradually release them, prolonging GF lifespan and enhancing GF action [40].

Finally, Francés-Herrero et al. used an EndoECM hydrogel that was obtained from pig uterus in an unpolymerized form to supplement human endometrial organoid culture medium, widening the range of possible uses and applications of this biomaterial [106].

### 3.2. Ovarian Hydrogels

The ovaries are the female gonads and play two important roles in the female reproductive system: they produce sex hormones—estrogens and progesterone—to regulate the menstrual cycle and they are responsible for oocyte growth and maturation. Only two studies have developed ovarian ECM (OvaECM) hydrogels. Buckenmeyer et al. used them as a follicle carrier in a murine model of premature ovarian failure (POF), and Chiti et al. created a support that was based on an OvaECM hydrogel for human and murine follicle growth in vitro [108,109].

Given its phylogenetic proximity, fragments or whole pig and cow ovaries have been the most used for decellularization techniques. The ovarian ECM of these species presents high protein conservation with respect to humans [108,115,116]. In addition, the ovaries have human-like size and anatomy and, in the case of cow, an analogous mono-ovulatory dynamic. However, several groups have already exploited the potential of human ovarian ECM in allogeneic and xenogeneic studies, both in vitro and in vivo.

Immersion and agitation either of the whole organ or of small tissue fragments is the most appropriate protocol for ovarian decellularization to generate OvaECM hydrogels. Rather than using enzymatic agents alone, it is best to use a combination of these agents and detergents, as detergents efficiently disrupt cell-nuclei and membranes and consequently promote better decellularization [108]. Perfusion of the whole ovary can be a very slow process and requires large volumes of detergents because of the complex anatomy of the blood vessels being cannulized. Perfusion and immersion are equally efficient at decellularizing the ovary [81], so the latter is preferred due to the ease of the procedure.

### 3.3. Oviductal Hydrogels

The oviducts are the anatomical structures connecting the ovaries and uterus and play a key role in reproductive processes. Fertilization takes place in these muscular tubes and, once embryo development begins, they generate contractions to transport the embryo to the uterus. For this reason, oviduct ECM (OviECM) hydrogels are attractive potential tools for studying early embryonic development.

In 2021, Francés-Herrero et al. generated a rabbit OviECM hydrogel, the only one reported to date in this field. The decellularization was performed via immersion and agitation of small oviduct pieces, and a modified version of the Freytes protocol was used to solubilize the lyophilized oviductal matrix [110]. The authors then generated coatings on culture plates by exposing the base of the wells to OviECM pre-gel solution overnight at 4 °C and washing it with phosphate buffered saline. Curiously, no specific decellularization protocols for obtaining whole OviECM scaffolds have been published. Isolating the oviducts from a uterine ECM scaffold after complete decellularization by perfusion would likely be the most efficient way to obtain a complete decellularized oviductal scaffold.

### 3.4. Vaginal Hydrogels

The vagina is a fibromuscular elastic tubular tract which connects the uterus and cervix to the outside of the body, allowing for menstruation, intercourse, and childbirth. The absence of this organ, due to congenital or acquired factors, can cause patients to suffer physical and mental pain and prevents normal sexual and reproductive processes [111].

Thanks to bioengineering technology, ECM products exist for vaginal reconstruction, such as vaginal ECM scaffolds [94,95] and hydrogels [111]. For vaginal ECM decellularization, several authors used the immersion protocol with chemical and enzymatic agents. However, prior (manual) removal of the mucosal and/or muscularis layers is important for better permeabilization of chemicals [94,111]. In the studies that have been carried out to date, the pig is the animal species of election par excellence given its phylogenetic proximity, the ease to obtain the raw material, and its similar female anatomy.

Hou et al. used a distinct strategy to create biomimetic 3D vaginal tissue using acellular vaginal matrix bioink. For this purpose, decellularized vaginal ECM was lyophilized, and solubilized, resulting in a vaginal ECM pre-gel. This pre-gel form was converted to bioink by adding gelatin and sodium alginate, which was then used to print 3D vaginal scaffolds. In addition, the authors proved the viability of these structures by in vitro encapsulation of bone marrow mesenchymal stem cells (BMSCs). The BMSCs had a high survival rate within the 3D scaffold, indicating the scaffold may allow unlimited nutrition and oxygen delivery to the cells, which makes it possible for long-term culture in vitro. Biocompatibility testing in vivo used transplantation of 3D scaffold-encapsulated BMSCs into rats, with positive outcomes [111].

### 3.5. Placental Hydrogels

The placenta is a temporary organ that allows communication between the mother and the fetus, transferring oxygen and nutrients from the former to the latter, and permitting the release of carbon dioxide and waste products from the fetus. It contains various physiologically active components, e.g., cytokines, GFs, and amino acids, and plays a unique role in feto-maternal tolerance, preventing the fetal “allograft” from being rejected. Therefore, amnion-derived material could be applied to reduce host immune response during allogeneic transplantation. The use of this human-derived tissue can greatly decrease the need for xenogeneic scaffolds [114].

Because it is challenging to obtain material (i.e., it requires a cadaver or disease-related excisions), no reports yet describe an attempt to develop ECM hydrogels from human uterus, ovary, or oviduct. However, the discarding of placental material postpartum enabled the development of human placental ECM hydrogels (Table 1).

Curiously, placental ECM hydrogels have not been applied to repairing or regenerating organs of the female reproductive tract, but have been used in organs and tissues of other systems [112,113,114]. There are two independent groups that have created human placental ECM hydrogels by immersion of ground or whole organs in detergent solutions and subsequent digestion and neutralization. These hydrogels could effectively support cardiomyocytes in vitro, and, when injected into ischemic myocardium, they reduced scar formation in vivo while maintaining electrophysiological activity [112]. The hydrogels also restored the hair-inducing properties of dermal papilla cells both in vivo and in vitro [113].

Mechanical delamination of the amniotic membrane from the fresh placenta followed by further decellularization is another strategy to obtain placenta-derived ECM hydrogels. Ryzhuk et al. generated a human amniotic membrane ECM hydrogel and showed that it could be recellularized by various types of stem cells. Amniotic membrane ECM hydrogels are, therefore, a promising material for use in tissue repair and regeneration due to their angiogenic nature, biocompatibility, and ability to biodegrade through a natural process [114].

## 4. Current Approaches in Bioengineering the Reproductive Tract Using ECM Hydrogels

**Figure 2:**
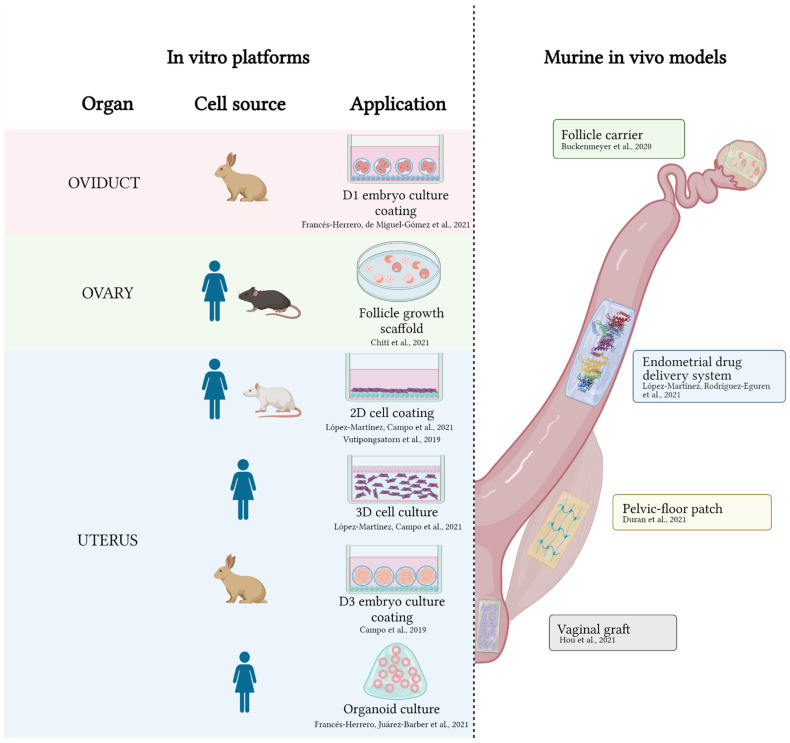
Applications of decellularized ECM hydrogels in the female reproductive tract. On the left, in vitro platforms involving ECM hydrogels from oviducts, ovary, and uterus. On the right, in vivo models where ECM hydrogels have been applied. References are indicated adjacent to each application [40,52,106,107,108,109,110,111,117,118]. Created with Biorender.com.

Applying tissue-specific ECM hydrogels to the development of in vitro platforms or in vivo models is considered the state-of-the-art at the convergence of bioengineering and biomedicine, given the powerful microenvironments that they provide. Yet, to date, few groups have employed them in the reproductive field, even though ECM hydrogels that are derived from reproductive tissues are described in the literature. An up-to-date list of all applications in animal models or using animal or human components of ECM hydrogels is presented in Figure 2.

### 4.1. Development of Next Generation In Vitro Platforms

#### 4.1.1. Coatings and 2D Cell Culture

In cell culture, culture plate coatings enhance cell attachment, migration, and functionality [119]. Tissue-specific coatings, rich in ECM components, GFs, and other molecules, improve these features [120]. For this reason, endometrial ECM (EndoECM) hydrogels warranted investigation as 2D culture coatings for endometrial cells. First, López-Martínez et al. produced a porcine EndoECM hydrogel that was able to be implemented as a coating for culturing human endometrial cells, including primary stromal and epithelial cells or stromal and epithelial stem cell lines [52]. EndoECM also served as a feeder for culturing these cells types in a 2.5 D structure and was compared to collagen coating or the synthetic ECM hydrogel Matrigel^®^, which cannot be used in the clinical routine given its tumorigenic origin and undefined chemical properties [121]. Cell viability measurements that were obtained through tetrazolium or live/death assays revealed no differences among groups but proliferation analysis by Ki67 immunostaining showed increased proliferation of cells in EndoECM. To test the biocompatibility and in vivo potential of EndoECM hydrogels, the authors injected the hydrogels into immunocompetent mice [52]. They demonstrated that their EndoECM contained a minimal amount of potentially immunoreactive molecules and was consequently hypoimmunogenic in vivo. This study proved that EndoECM hydrogels can improve cell proliferation, positioning them as an alternative coating for use in routine cell culture protocols, as well as a suitable candidate to be applied in vivo.

EndoECM hydrogels that are produced from rats also show some success. Vutipongsatorn et al. cultured rat endometrial stromal cells in a rat EndoECM hydrogel and measured their viability by a live/dead assay at two, four, and seven days. They found that their EndoECM could host living cells [117], consistent with the López-Martínez et al. study.

Campo and colleagues were innovators in applying tissue-specific ECM hydrogels as coatings for culturing embryos [107]. They studied two EndoECM hydrogels of rabbit-origin, testing whether the cycle phase at which the endometrium was decellularized affects embryo culture. To achieve this, they obtained non-synchronous endometria with non-proliferative endometrium (NS) and synchronous endometria after ovarian stimulation with a GnRH agonist (S). EndoECM was obtained and used for plate coating, and day-three early blastocysts/late morulae were cultured on S, NS, or Matrigel (M). Coating with the S hydrogel, but not the NS hydrogel, yielded a similar hatching/hatched embryo rate to that of Matrigel and similar patterns in expression of stem cell markers (OCT4 or SOX2). This work demonstrated the relevance of mimicking the endometrial environment in vitro to achieve suitable embryonic development, as a synchronous endometrium is needed for embryo implantation.

OviECM offers another source of material that could be used to create hydrogels for embryo culture at earlier time-points, as the oviduct is the natural site of fertilization and where preimplantation embryos develop [110]. To test this, two-cell rabbit embryos were cultured on rabbit OviECM coatings or in standard medium. The embryos in both groups had similar morphology and developmental dynamics, i.e., both reached late morula/early blastocyst stage in a comparable time-frame (48 h). Metabolic analysis of the media with or without embryos was performed for both experimental groups. The composition of the media from embryos that were cultured on the OviECM was similar at the start and end-point of the culture period [110]. This matches the “quiet embryo hypothesis”, which claims that viable embryos present a ‘quieter’ metabolism than non-viable ones [122]. Their work confirms that hydrogels retain bioactive ECM-specific proteins in their structure and slowly release them into the cell culture medium [110].

Recently, Chiti et al. designed decellularized OvaECM hydrogels that are able to support human and murine ovarian follicle survival and growth in vitro [108], which is also described in ovarian ECM scaffolds [82]. Even if OvaECM hydrogels provide more suitable microenvironment than native scaffolds, based on rheological characterization, the hydrogel exhibited lower rigidity than the native ovary. For this reason, the OvaECM hydrogel was combined with alginate to obtain hybrid hydrogels, which provided the necessary consistency to allow the follicles to expand [108]. To define the best concentration of alginate, the authors analyzed the follicle recovery rate, viability, and growth in 100% OvaECM, 90% OvaECM–10% alginate, 75% OvaECM–25% alginate, and 100% alginate. They found that increasing the alginate concentration caused a proportional increase in the follicle recovery rate, but most conditions had similar follicle viabilities and diameters. Relative to the other groups, only the 100% OvaECM group exhibited no follicle recovery and had differences in viability and follicle diameters. In spite of showing better results, pure alginate is not apt to be ultimately applied in the ovary, given its low degradability and poor vascularization after transplantation [108]. This study describes the potential use of synthetic materials to create hybrid hydrogels that have the appropriate stiffness that is needed to maintain specific 3D structures with improved biofunctionality.

#### 4.1.2. 3D Cultures and Organoids

Two-dimensional cultures have limited ability to mimic the natural microenvironment, but 3D cultures are designed to simulate the interaction, proliferation, and differentiation stimulated by in vivo tissue-specific conditions. To overcome that issue, some authors developed 3D structures using reproductive ECM scaffold that was filled with diverse cell types, as reported from uterus [51,64,69,71,72,73], ovary [83,85], vagina [94,95], or placenta [97] scaffolds. However, current 3D culture conditions usually lack cell-ECM interactions, which are essential to reproduce physiological conditions [123]. López-Martínez et al. demonstrated an implementation of their porcine EndoECM cell coating as a 3D co-culture (epithelial and stromal) system for endometrial primary and stem cells [52]. They compared this approach to standard culture conditions and, after 10 days, found no difference between the two groups in terms of cell proliferation. Cells that were cultured with EndoECM had normal phenotype and expressed typical markers of endometrial cells. Moreover, cells in the EndoECM group were more proliferative. The EndoECM also became reorganized over time, reducing in volume. The phenomenon occurs for other collagen-based hydrogels [124].

This same EndoECM was the first to be used as a supplement for human endometrial organoid culture [106]. Organoids are self-assembled 3D multicellular in vitro structures that better mimic their organ of origin than standard 2D cell cultures [125]. Organoids that are derived from human endometrial biopsies were grown in Matrigel and cultured in four different conditions: the optimal standard medium (ExM), suboptimal media lacking nicotinamide (ExM-NA), and these mediums that were supplemented with pre-gel solution of EndoECM (ExM + EndoECM and ExM-NA + EndoECM) at 0.1 mg/mL. Spheroid counting revealed that EndoECM supplementation enhanced proliferation with respect to ExM and was able to overcome the lack of NA. Moreover, EndoECM did not disrupt the glandular epithelial features of the organoids based on marker analysis. The organoids expressed epithelial markers (cytokeratins), lacked stromal markers (vimentin), and retained progenitor cell markers (N-cadherin and SSEA-1). Further, there were no long-term chromosomal alterations in cells that were cultured with EndoECM. Overall, EndoECM supplementation provided a promising environment for organoid development, complementing ExM medium and the use of Matrigel, which does not fully recapitulate the in vivo dynamic milieu. However, a limitation of this study was the use of EndoECM as part of the culture medium rather than as a substitute for Matrigel, given its ability to gel. A study of intestinal organoids did find that substituting other collagen gels for Matrigel stimulated organoid self-organization into centimeter-long tubes in the floating gel [126].

### 4.2. In Vivo Applications: Animal Models

#### 4.2.1. Carriers for Cell Transplantation

In situ cell delivery may improve treatments for diverse diseases. Compared to direct administration of cells or integration in ECM scaffolds [89,98], the use of hydrogels as carriers can enhance the rates of cell retention and cell engraftment in vivo [127]. Buckenmeyer patented an ovarian-derived hydrogel (OvaECM) for biomedical and biotechnology applications (US 2017/0252485 A1) and implemented it as a delivery system of follicles in a murine model of chemotherapy-induced POF [109]. Follicles were obtained from mouse donors, labeled with GFP, mixed with OvaECM, and carefully microinjected into the ovaries of recipient females. A total of two weeks after injection, these mice were mated with males through three breeding cycles. The mice produced healthy offspring, demonstrating suitable engraftment of the follicles. This technique appears to be a minimally invasive option to restore the ovarian reserve post-chemotherapy, and seems less risky than using ovarian cortex transplantation, which despite being promising, entails the potential introduction of malignant cells into the body [128].

#### 4.2.2. Patches

Some groups employed reproductive ECM scaffolds as patches [67,68,77,79]. However, they offer the disadvantage of being applied invasively and they lack intense biochemical interaction with the transplantation site. In contrast, ECM hydrogels are minimally invasive systems that can be introduced with a syringe and they conform to the shape of the defect site [55]. Moreover, exposure to cytokines, chemokines, GFs, cryptic peptides, bioactive motifs, and bioactive matrix-bound nanovesicles from hydrogels can affect surrounding cell behavior and, therefore, enhance wound healing and cell differentiation, even if the regenerative effect of ECM hydrogels is not yet totally understood [55]. Accordingly, ECM hydrogels can serve as patches for regeneration in the reproductive tract. Duran et al. patented a porcine skeletal muscle ECM hydrogel (SKM) to treat pelvic floor disorders (WO 2018/213375 A1). This biomaterial was applied in a rat model of simulated birth injury (SBI) to assess its efficacy as a pro-regenerative scaffold [118]. Briefly, after inducing and validating the SBI model, SKM or saline was either injected immediately or four weeks later after birth, with the latter injection designed to mimic the timing of a routine post-partum visit. Tissues were harvested four weeks after SKM injections. Compared to the untreated or saline-injected SBI mice, hydrogel-injected SBI mice had substantially reduced collagen deposition and increased fiber areas that were similar to those of uninjured rats. However, vascularization did not vary significantly by treatment. Over the long-term, SKM potentiated muscle recovery and even induced greater fiber area than that of the uninjured control and decreased the fibrotic response. Transcriptional studies revealed a decrease in the expression of several pro-inflammatory genes. Consequently, this research suggests a novel approach to treat SBI with a minimally-invasive acellular biomaterial.

ECM hydrogels can be bioprinted into 3D constructs, but, in the reproductive field, this has only been reported for the vagina. Hou et al. produced a 3D graft that was made by a bioink from solubilized vaginal ECM, which was used to encapsulate BMSCs. It was introduced into subcutaneous pocket in rats to evaluate its functionality relative to a bioink 3D graft without BMSCs [111]. A total of four weeks after grafting, 3D vaginal tissues had successfully implanted and exhibited progressive vascularization, epithelization, and cell differentiation in the BMSC group. Note that the regenerated epithelium seemed immature, probably due to the short implantation time in the experiment. This study suggests an innovative and promising alternative for vaginal reconstruction or even vaginal substitution; however, further research is required.

#### 4.2.3. Drug Depots

ECM hydrogels also offer the possibility of acting as drug delivery vehicles given their hydrophilic and highly porous nature that can accommodate the controlled release of substances [129]. Recently, López-Martínez and collaborators applied their porcine EndoECM as a carrier of growth factors to revert a murine model of endometrial damage [40]. A biotin-labelled EndoECM was supplemented with 10 ng/mL basic fibroblast growth factor, 100 ng/mL platelet-derived growth factor-BB, and 100 ng/mL insulin-like growth factor-1, and its regenerative effect was compared to that of EndoECM alone and a saline negative control. The treatments were applied four days after endometrial ethanol-induced damage, and two weeks later, tissue regeneration was examined, and fertility assessed by natural mating. The supplemented EndoECM group exhibited a decrease in a marker of chronic inflammation (i.e., collagen deposition) and an increase in the number of glands, endometrial area, neovasculogenesis, and cell proliferation compared to the saline group. Consistent with this, the number of embryos that were produced by the supplemented EndoECM group was similar to that of undamaged animals. This is likely due to the ability of ECM fibers to carry and keep biomolecules, e.g., GFs, and gradually release them prolonging their lifespan and enhancing their action [40,52]. Thus, this hydrogel may optimally release biological products, thereby stimulating better tissue regeneration than EndoECM alone.

### 4.3. Application of Non-ECM Hydrogels in the Human Reproductive Tract

Therapeutic application of ECM hydrogels remains restricted to animal models. Thus, in this section, we present the major natural, synthetic, or hybrid non-ECM hydrogels that have already been applied in the field of women’s reproductive medicine (Table 2) that may pave the way for ECM-based translation. Collagen scaffolds have been mainly used in clinical trials to carry stem cells, e.g., umbilical cord mesenchymal stem cells (UC-MSC), to effectively treat thin endometria or endometria with adhesions [130,131]. In order to build the UC-MSC/collagen complex, both groups used a 4 cm × 6 cm porous collagen scaffold with a porosity of 20–200 µm of diameter. The cell-seeded scaffold was incubated for an hour prior to its transplantation. Cao et al. attached the complex to the endometrial wall for 12 h while Zhang et al. left it for three days. Collagen was also used as a carrier of basic fibroblast growth factor (bFGF) and this treatment successfully achieved repair of adhesions in patients with Asherman syndrome [132]. Similarly, Jian et al. employed a collagen-binding domain to gradually release bFGF in the endometrium. To highlight, the three groups demonstrated that their treatment significantly improved endometrial thickness and the expression of different regenerative markers such as Ki67, estrogen receptor α, or progesterone receptor was increased. More importantly, the live birth rate improved in the treated women, demonstrating the effectiveness of collagen-derived treatments to cope with atrophic endometria.

Collagen-based carriers have also been employed to treat ovarian defects. Ding et al. used a novel scaffold of UC-MSCs to activate follicles in patients with POF [133]. Briefly, they mixed degradable collagen fibers with UC-MSCs and injected it into the ovary unilaterally under transvaginal ultrasonography. The authors described the restoration of the ovarian function by the presence of mature follicles in some patients, which eventually allowed successful clinical pregnancy.

Sprayable hydrogels show high adhesiveness to wound surfaces and are minimally invasive. In the female reproductive tract, hydrogels that were made from a synthetic polyethylene glycol (PEG) derivative [134,135] or combined with dextran aldehyde (Actamax^TM^ Adhesion Barrier) [136] have been reported in spray format to avoid the formation of adhesions after myomectomy. These hydrogels were sprayed into surgical traumas and adjacent structures using gas-assisted systems. They all led to the significative reduction of adhesions that were derived from myomectomy and the safety of the products was highlighted. These types of hydrogels are easy-to-apply, either in laparoscopy or open procedures, and can prevent postoperative adhesions.

## 5. Future Perspectives

The application of bioengineering techniques in the field of reproductive medicine is not new. A multitude of natural, synthetic, and hybrid materials, either in the form of hydrogels or solid scaffolds, have been used in vitro, in animal models, and in clinical trials. Decellularization techniques to obtain ECMs from reproductive organs are increasingly important to advance the field, and diverse protocols support their development for virtually all tissues and organs of the female reproductive tract. However, the use of these matrices in their solubilized and neutralized form remains in its infancy in women’s reproductive medicine and is almost non-existent for treating reproductive defects in men [137,138].

Most studies reporting the use of ECM hydrogels in the reproductive field do so in an in vitro context. There is evidence that these biomaterials have potential in the functionalization of culture surfaces or as substrates for 3D culture. Research in this area might not only improve current culture platforms and in vitro models of gynecological diseases but could also represent a paradigm shift in assisted reproduction clinics. The culture of human follicles and embryos may benefit from these biomaterials, as some preclinical studies suggest [106,108].

As has been demonstrated in clinical areas such as cardiology [21] or in murine models of gynecological diseases, ECM hydrogels could expand the in situ therapeutic options for addressing diseases such as POF, Asherman syndrome, or endometrial atrophy [40,109]. Using hydrogels as carriers for cells, drugs, or other biological derivatives is nearly a reality. Moreover, advances in reproductive medicine could be transferred to other clinical areas as demonstrated by the utility of placental ECM hydrogels.

Finally, as demonstrated by Hou et al., the use of ECM hydrogels in the field of reproductive medicine could be combined with other bioengineering techniques such as microfluidic systems or 3D bioprinting (as bioinks) [111,139,140], generating complex in vitro systems and transplantable 3D constructs.

If ECM hydrogels consolidate as a reference biomaterial in the field of reproductive medicine and research, the availability of female reproductive organs for mass production could be a potential limitation. However, the use of xenogeneic approaches would facilitate the procurement of raw material, preventing this from being a restriction to the expansion and further development of ECM hydrogels. In addition, several studies have demonstrated the almost universal compatibility of ECM matrices among mammals, becoming one of the main strengths of these biomaterials [47,48].

Finally, note that with the rapid progress in bioengineering techniques, it is to be expected that ECM hydrogels will be increasingly refined and adapted to the specific requirements or needs of different applications. In this sense, purifying certain components of the matrices, or enriching the hydrogels with molecules or factors of interest will enhance the desired performance.

## 6. Conclusions

In conclusion, reproductive medicine has benefited from many advances in the development and application of natural and synthetic biomaterials. Due to the quest to better mimic the native microenvironment in which different physiological processes take place, there is increasing interest in tissue-specific decellularized ECM. Hydrogels that are created by the solubilization of tissue-specific ECM have been developed, characterized, and widely applied in virtually all medical disciplines, but have only been timidly adopted in the field of human reproduction. Promising in vitro studies and animal models highlight the potential of these hydrogels, but future studies are required for clinical applications.

## Figures and Tables

**Table 1:** Obtaining ECM hydrogels from the female reproductive tract. The tissue of origin, decellularization protocol, characterization techniques, and main findings of the studies are indicated.

Organ	Species	ECM Hydrogel	Decellularization Protocol	Characterization Techniques	Main Findings	Reference
**Uterus**	Rabbit	Endometrium	Perfusion; chemical and enzymatic (48 h).	HIS, PRO, SEM, TURB	Coatings of EndoECM hydrogels at different phases of the oestrous cycle influenced in vitro embryo development in a rabbit model.	[107]
	Pig		Perfusion; chemical and enzymatic (48 h).	DNA/Collagen/Elastin/GAGs quant, HIS, IHC, MTS assay, PRO, SEM, TURB	Porcine EndoECM hydrogels supported 2D and 3D in vitro culture of human endometrial cells and developed a hypoimmunogenic reaction in vivo.	[52]
					Injection of EndoECM hydrogels alone or supplemented with growth factors repaired the endometrium and restored fertility in a murine model of endometrial damage.	[40]
					Supplementation of the culture medium with EndoECM hydrogels enhanced the proliferation potential of human endometrial organoids.	[106]
**Ovary**	Cow	Ovarian fragments	Agitation; two different protocols: (I) enzymatic (38 h); (II) chemical and enzymatic (24 h).	DNA/Collagen/GAGs quant, REO, SEM	An OvaECM-based hydrogel supported human ovarian follicle survival in vitro.	[108]
	Pig		Agitation; chemical and enzymatic (5 days).	DNA/GAGs/HYP/Protein quant, ELISA, HIS, IHC, REO, SEM, TURB	Encapsulation and delivery of murine follicles in an OvaECM hydrogel yielded offspring in a chemotherapy-induced POF mouse model.	[109]
**Oviduct**	Rabbit	Whole oviduct	Agitation; chemical and enzymatic (48 h).	DNA/GAGs/HA quant, HIS, PRO, SEM	In vitro culture of rabbit embryos on an OviECM hydrogel coating induced a quiescent metabolism that better mimicked the physiological state.	[110]
**Vagina**	Pig	Whole vagina	Agitation; chemical and enzymatic (10 days).	DNA quant, HIS, REO, SEM	Acellular vagina matrix bioink supported bone marrow mesenchymal stem cell growth in vitro and promoted vascularization, epithelialization, and cell differentiation in vivo.	[111]
**Placenta**	Human	Chorionic plate	Agitation; chemical (24 h).	ELISA, MS, SEM	A placental ECM hydrogel effectively supported in vitro cardiomyocyte culture and reduced scarring in a rat model of cardiac ischemia.	[112]
		Whole placenta		HIS	Human placenta ECM hydrogel-cultured dermal papilla spheres co-grafted with new-born mouse epidermal cells regenerated new hair follicles.	[113]
		Amniotic membrane	Agitation; physical, chemical and enzymatic (24 h).	Collagen/Elastin/GAGs/Protein quant, HIS, SEM, TURB	Development of human amniotic membrane ECM hydrogel that supported the proliferation of various stem cell types and induced low inflammatory reaction in vivo.	[114]

Abbreviations: ELISA: enzyme-linked immunosorbent assay; EndoECM: endometrial extracellular matrix; GAGs: glucosaminoglycans; HA: hyaluronic acid; hECs: human endometrial cells; HIS: histology; HYP: hydroxyproline; IHC: immunohistochemistry; MS: mass spectometry; MTS: 3-[4,5,dimethylthiazol-2-yl]-5-[3-carboxymethoxy-phenyl]-2-[4- sulfophenyl]-2H-tetrazolium, inner salt; OvaECM: ovarian extracellular matrix; OviECM: oviductal extracellular matrix; POF: premature ovarian failure; PRO: proteomics; qPCR: quantitative polymerase chain reaction; quant: quantification; REO: rheology; SEM: scanning electron microscopy; TEM: transmission electron microscopy; TURB: turbidimetry.

**Table 2:** Non-ECM hydrogel application in human clinical trials.

Type of Hydrogel	Material	Organ	Application	Main Outcomes	CT Number	References
**Natural**	Collagen 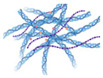	Uterus 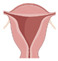	Carrier of stem cells	Increased endometrial thickness, proliferation, differentiation and neovascularization. Achievement of pregnancy.	NCT02313415	[130]
				Increased endometrial thickness, angiogenesis, proliferation and response to hormones. Achievement of pregnancy.	NCT03724617	[131]
			Carrier of growth factors	Increased endometrial thickness, menstrual blood volume, scarring regeneration. Achievement of pregnancy.	ChiCTR-OPC-17010786	[132]
		Ovary 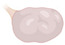	Carrier of stem cells	Rescued ovarian function, improved follicle development and antral follicles. Achievement of pregnancy.	NCT02644447	[133]
**Synthetic**	PEG 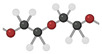	Uterus 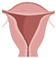	Tissue regeneration	Reduction of adhesions after myomectomy.	NCT00562471	[134,135]
**Hybrid**	PEG + Dextran 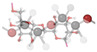	Uterus 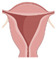	Tissue regeneration	Reduction of adhesions after myomectomy. Easy to administer.	NCT02260115	[136]

Abbreviations: CT: clinical trial; ECM: extracellular matrix; PEG: polyethylene glycol.

## Data Availability

Not applicable.

## Supplementary Materials

The following supporting information can be downloaded at: https://www.mdpi.com/article/10.3390/ijms23073765/s1.
